# Food Insecurity in the Post-Hurricane Harvey Setting: Risks and Resources in the Midst of Uncertainty

**DOI:** 10.3390/ijerph17228424

**Published:** 2020-11-13

**Authors:** Kevin M. Fitzpatrick, Don E. Willis, Matthew L. Spialek, Emily English

**Affiliations:** 1 Department of Sociology and Criminology, University of Arkansas, Fayetteville, AR 72701, USA; 2 Department of Internal Medicine, University of Arkansas for Medical Sciences, Fayetteville, AR 72701, USA; dewillis121611@gmail.com; 3 Department of Communications, University of Arkansas, Fayetteville, AR 72701, USA; mspialek@uark.edu; 4 Department of Pediatrics, University of Arkansas for Medical Sciences, Fayetteville, AR 72701, USA; esenglish@uams.edu

**Keywords:** food insecurity, natural disasters, risks and resources, hurricane Harvey

## Abstract

Food insecurity is of heightened concern during and after natural disasters; higher prevalence is typically reported in post-disaster settings. The current study examines food insecurity prevalence and specific risk/resource variables that may act as barriers or advantages in accessing food in such a setting. Using a modified quota sample (*n* = 316), Hurricane Harvey survivors participated in face-to-face interviews and/or online surveys that assessed health, social and household factors, and sociodemographic characteristics. Using logistic regression analyses we find that social vulnerabilities, circumstantial risk, and social and psychological resources are important in determining the odds of food insecurity. Hispanic and/or Nonwhite survivors, renters, and those persons displaced during the natural disaster have higher food insecurity odds. Survivors with stronger social ties, higher levels of mastery, and a greater sense of connectedness to their community are found to have lower food insecurity odds. A more nuanced analysis of circumstantial risk finds that while the independent effects of displacement and home ownership are important, so too is the intersection of these two factors, with displaced-renters experiencing significantly higher odds than any other residence and displacement combinations, and particularly those who are homeowners not displaced during the disaster. Strategies for addressing differential risks, as well as practical approaches for implementation and education programming related to disaster recovery, are discussed.

## 1. Introduction

Despite an abundance of food, over 37 million—roughly one in ten—people in the U.S. lack “consistent access to healthy and adequate amounts of food for an active and healthy life” [1]. Scarcity of food cannot explain hunger and food insecurity globally, particularly in the food abundant U.S. [2,3,4]. Thus, explanations of food insecurity demand a social science lens that focuses attention on how food is distributed across social and hierarchical lines. For example, the prevalence of food insecurity jumps from one in ten in the general U.S. population to one in three in low-income U.S. households with children [1]. It is not surprising that poverty is a leading determinant of food insecurity; still, this challenges the “productivist” notion that food insecurity is the byproduct of food scarcity [5].

Scarcity as a justification for food insecurity is not the only way that humans “naturalize” its existence—thinking of it as the “natural” consequence of forces outside our social, political, or otherwise collective control. For example, we often think of hunger and food insecurity in the wake of extreme weather events to be a natural consequence, almost giving agency to the event itself—we even name them! While extreme weather may be—to some degree—outside our immediate collective control, responses to (e.g., plans for the distribution of emergency resources) and the severity of who is impacted is not. The emphasis on the “natural” elements of disasters is myopic and masks an important set of underlying social dimensions. From this narrow view, an event such as a hurricane is merely a meteorological phenomenon with natural consequences—few questions are raised about whether the event had to be as catastrophic to human lives as it was, or why certain groups tend to face more catastrophe than others during and after the same event.

The present study challenges the naturalization of both food insecurity and disaster with specific interest in the differential experience of food insecurity in a post-disaster setting and the unnatural social conditions that might lead to those unequal experiences. In doing so, we ask the very questions that the naturalistic view would not: (1) Who is at risk and who is protected from food insecurity in a post-disaster setting; (2) which specific risks and resources—rooted in structural and individual circumstance—matter most in this setting?; and, (3) how do risks such as displacement and not owning a home interact with one another? These questions force us to consider ways in which post-disaster food insecurity is socially determined, rather than leaving the unequal consequences to be considered a natural or taken-for-granted result. Our analysis also assesses the role of many factors shown previously to be associated with food insecurity outside of natural disaster scenarios or settings, such as age, gender, race, income, the presence of children in the household, and home ownership [1,6,7,8,9].

### Disaster and Food Insecurity

Eric Klinenberg’s [10] seminal work offers a valuable strategy for analyzing natural disasters: A “social autopsy” which takes inventory of the social conditions that make disasters worse, and for whom. His research demonstrates that natural disasters are not exposing/impacting everyone uniformly. There are combinations of relative risks and resources which shape where and for whom catastrophe is worsened or mitigated. Moreover, the risks and resources which differentiate the experiences of disasters are deeply rooted in our social structure [10,11].

Just like the experience of the initial disaster, the lasting impact and disaster recovery is not a uniform experience for all survivors; in particular, the ability to maintain consistent access to enough healthy food needed for an active and healthy life is not equally constrained or enabled across the impacted population. An estimated seven million individuals impacted by Hurricane Harvey in 2017 were considered to be food insecure, and those persons most vulnerable and already at risk prior to any natural disaster were concentrated in particular population subgroups [12]. It is precisely this expected inequity that we explore in the current study. We analyze vulnerabilities that are linked to social structure, as well as individual-level risks and resources often associated with food insecurity. We explore these relationships among survivors following Hurricane Harvey, one of the most catastrophic weather events in recent memory. In doing so, we reveal some of the unnatural factors shaping food insecurity in the aftermath of this disastrous hurricane.

How and where to obtain healthy food can become a complex puzzle in post-disaster communities, struggling to put back the pieces of their life that were torn apart after physical and emotional devastation. A once active food pantry system can become fractured, and service providers like the Red Cross, Salvation Army, and local churches are often left scrambling to fill in the gap until reliable, consistent food access can be restored. In some cases, like the recent devastation in the Bahamas brought on by Category 5 Hurricane Dorian, extraordinary efforts were required in order to create emergency feeding systems. These systems tend to rely on random volunteers who are mobilized because service providers are often overwhelmed in their own recovery and have difficulty getting food out the door to survivors who have lost everything [13]. Food supply chains are disrupted, resources are depleted, and the availability of reasonably priced food is no longer in abundance. In some cases, it may take weeks or months to fully restore the underlying infrastructure that residents rely on for consistent access to healthy foods [14].

These complicated circumstances beg the questions: What about food insecurity among vulnerable, post-disaster populations? Are there groups of survivors more at risk than others because of certain structural vulnerabilities that are present before disasters hit? What individual-level risk and resource factors are related to food insecurity in post-disaster settings? The current study examines these questions on food insecurity in the post-disaster Texas Gulf Coast after Hurricane Harvey made landfall on 22 August 2017.

While there are studies examining some of these factors [15,16,17,18], the current study looks to go beyond some of this work and examine levels of vulnerability, risk, and resources in order to develop and implement specific food assistance programming that can be supported and sustained through weeks and months after a disaster. Knowing who is at risk and what factors might help to mitigate those risks can be important to developing and targeting programs in particular communities where residents are considered to be high risk/vulnerable. Understanding the link between these risks and social structure will be critical to the development of long-term, sustainable solutions that equitably protect against the unnatural (i.e., avoidable) consequences of natural disasters. Moreover, our questions also consider the potential interactive effects of displacement and renting and how that may differ compared to those owning their place of residence.

## 2. Theory and Evidence

### 2.1. The Disaster Setting

Not unlike other disasters, hurricanes can cause widespread destruction through immediate and long-term impacts. The immediate disruption takes place through storm surge, with high tide and destructive winds, while the longer-term impacts of a hurricane can occur through the rainfall and subsequent flooding. In August of 2017, Hurricane Harvey devastated Houston—the fourth largest city in the United states—and its surrounding areas with record-breaking rainfall amounting to approximately 60 inches [19,20]. The category 4 hurricane was the largest to hit Texas in over half a century [21,22].

This disaster was familiar to many, partly because its impact was very similar to that of Hurricane Katrina, which hit the Gulf Coast of Mississippi, Alabama, and Louisiana in August 2005. The survivors of Katrina, that bore the brunt of that devastation, were disproportionally low-income, older, and African-American. Katrina and the work that followed over the next several decades, put a spotlight on the notion of subgroup vulnerability and the varying individual-level risks and social and psychological resources that made a difference in both individual and community recovery [16,23,24].

What many had witnessed in New Orleans, and in other places, was that competing demands to obtain safety and shelter exacerbated precarious life circumstances for certain groups that were either on the cusp of experiencing food insecurity or were already food insecure. Disasters often reveal vulnerabilities in communities where individuals are already at risk, and the disruption of informal social networks and access to formal service provision can have devastating consequences on long-term, sustainable food sources. Displacement can disrupt support, it can disrupt daily routine and elevate stress, and it can be responsible for negative health and well-being outcomes that are manifested both physically and mentally. An examination of how that ecosystem of support is disrupted and damaged is vital to the social autopsy that Klinenberg [10] discusses.

In an effort to better understand these varying vulnerabilities and differences in risks and resources across populations in a disaster setting, the current study utilizes a risks and resource framework to examine how food insecurity may have been intensified or exacerbated by a natural disaster. A risks and resources framework can be useful in determining the relationship among risks that negatively impact food insecurity and what, if any, social and psychological resources could help to mitigate risks for food insecurity [25]. This risks and resources approach is somewhat distinct but complimentary to Klinenberg’s social autopsy; borrowing concepts from both allows us to consider specific ways in which risks and resources are connected to larger social structures while also providing a useful framework for explaining why some groups are more vulnerable to certain health consequences, like limited access to healthy food in post-disaster settings, while others experience more protection.

### 2.2. Social Vulnerabilities

With the anticipation that some social groups are more vulnerable than others to the health risks related to disaster, we explore some of these characteristics and their relevance in the Hurricane Harvey disaster setting. Social vulnerabilities arise when the risks and burdens of disastrous events are unequally felt across social groups and that disproportionate burden is linked to social forces. Examples of this include the disproportionate exposure to hotter neighborhoods felt by low-income and minority communities—an outcome linked to a history of systemic racism in housing policies, such as redlining [26].

Another example is the social isolation that puts elderly populations—especially among racial minorities—at increased risk for dying alone [10,27,28]. The impact of racial and ethnic segregation has also resulted in minorities occupying more low-lying, flood-prone, and amenity-poor places than their white counterparts [29]. Reports after Hurricane Harvey confirm this unequal exposure as flooding was considerably worse in areas with a larger Hispanic population when compared to White residents [30]. Compounding the physical exposure to Hurricane Harvey, Hispanic residents were also more likely to experience negative economic consequences post-disaster. Specifically, Hispanics were significantly more likely to report that they had experienced employment disruptions in the first few months following the hurricane when compared to White residents [31]. Inequalities that exist prior to the disaster also shape the ways in which social groups are able to respond and cope post-disaster. Prior to Hurricane Harvey, Houston was ranked the most economically segregated city in the United States [32]. Socio-economic status (SES), in particular, plays a significant role in shaping individuals ability to cope in the post-disaster setting [33]. The impact on health outcomes is also disproportionately felt across social groups. For example, while women tend to live longer than men, natural disasters lower the life expectancy of women more than men [34].

Given the findings of this body of literature, we expect significant differences in reported food insecurity between certain sociodemographic groups. Racial and ethnic minorities, lower-SES and women often experience the aftermath of natural disasters differently than their counterparts—in part because of the already difficult circumstances that many of them are living in, the places where they are living, and the limited access to resources they experience that are often exacerbated by natural disasters. As such, *we hypothesize that these socially and economically disadvantaged groups will have higher odds of food insecurity compared to their older, white, non-Hispanic, male, wealthier, and families without children counterparts.*

### 2.3. Circumstantial Risk

Natural disasters create many additional stressors that are associated with food insecurity, either directly or indirectly. Some examples of additional stressors that can be experienced as a result of a disaster are displacement, damage to property, physical and mental health strains, and financial loss [35]. In the disaster literature, displacement has not received much attention regarding its impact on food insecurity for survivors. Displacement disrupts social networks, sources of medical care, and access to social services [16,17,36]. Closely related to the concept of displacement are relocations. Researchers found that the number of moves a person made following Hurricane Katrina was significantly and positively associated with food insecurity [17]. More recently, Clay and colleagues [15] examine relocation and find that those persons who relocated regardless of where they went, experienced more food insecurity compared to those residents who did not leave their residence regardless of the damage experienced. Building on this literature related to displacement, relocation, and food insecurity, we hypothesize those persons *leaving their residence prior to or during the storm will report higher odds of food insecurity than persons who stayed behind in their residence.*

An additional circumstantial risk that we consider is residential status. Homeownership is closely tied to wealth and socioeconomic security in the United States, making it in many ways a proxy for social class. Furthermore, renters are often at the mercy of their landlords when it comes to the decision to vacate an unsafe living situation or remain in a unit to avoid the challenge of finding another affordable option in a city where affordable living is scarce [21,37,38]. This means that renting brings with a combined disadvantage of less wealth generation and less control over the decision to stay or leave during a disaster, placing renters at increased risk in emergency situations. Thus, *we expect renters will report higher odds of food insecurity than homeowners.* Furthermore, given the legal system, which allows landlords significant control in determining whether tenants can or must vacate during a natural disaster, *we expect to find a significant interaction between displacement and renting rather than displacement and home ownership.*

### 2.4. Social and Psychological Resources

The risks and resources framework is based upon the central assumption that certain resources can protect, or shield, individuals from negative risks and/or outcomes. Psychosocial resources can be both clearly social (e.g., social ties, community connectedness) and psychological resources (e.g., mastery) that are interconnected with social structures and social positioning.

A social resource shown to protect against negative health outcomes is an individual’s strength of social ties. The strength of social ties scale, developed by Lin and colleagues [39], determines the strength of an individual’s social connections with higher social support significantly reducing negative consequences on an individual’s health caused by stress [40,41,42,43,44,45]. In their recent work on food insecurity post-Hurricane Harvey, Clay and Ross [15] explore the role of capital and social ties as critical protective factors and find significant support for these variables and their role in mitigating the negative impact of disaster on food insecurity among hurricane survivors. As such, *we hypothesize that persons with higher perceived social ties will report lower odds of food insecurity compared to those with lower levels of social ties.*

We also consider the psychosocial personal coping resource of mastery of fate. Mastery has been conceptualized by some scholars as an indicator of agency [46]. As a determinant of agency, Thoits [46] argues that mastery is one of several personal coping resources which aids individuals in what Wheaton [47,48] called “stress deterrence”. Given this literature on the link between mastery and coping during stressful events, we *hypothesize that persons with higher levels of mastery of fate will report lower food insecurity odds compared to those with lower levels of mastery of fate.*

Community connectedness has been defined as a “feeling that members have of belonging, a feeling that members matter to one another and to the group, and a shared faith that members’ needs will be met through their commitment to be together” [49]. In other words, it is both a psychological and material resource—a link between belonging and a sense that one’s needs can and will be met. It is conceptually similar to social capital—the idea that membership in a social group acts as a “credential” which entitles members to “credits” [50]—which has been shown by some researchers to be a significant predictor of hunger and food insecurity [51,52,53]. There are three main types of social capital that all reflect different forms of community connectedness. Bonding social capital refers to close associations with homogenous or relatively like-minded individuals like family, friends, and neighbors. Bridging social capital encompasses connections that “span social groups, such as class or race,” [54]. Finally, linking social capital describes an individual’s perceived connectedness to key decision makers in a community. As such, *we expect people who perceive greater connectedness to their community will report lower odds of food insecurity compared to those with less perceived connectedness to their community.*

## 3. Data and Methods

### 3.1. Participants and Procedure

This study is based on data collected in Fall 2017, which generated a quota sample of 316 interviews with Hurricane Harvey survivors. A final analytical sample of 251 was included in the regression analyses after cases with incomplete information are excluded. While missing data are certainly a limitation, we argue this is mostly attributable to the difficult circumstances in which the data were collected and that the significance of the results outweigh this particular limitation. 

Individuals were recruited from locations Federal Emergency Management Agency (FEMA) determined as those with the highest damage estimates, which included counties of Brazoria, Galveston, Harris, Jefferson, and Nueces. To obtain a representative sample, each county’s total population estimates were determined and the largest cities within counties were selected for targeted sampling. A percentage of participants to be targeted for selection from each city was determined by comparing the overall percentage of persons directly or indirectly impacted by Hurricane Harvey according to FEMA. Of those that were targeted, the goal was to obtain interviews from an even gender distribution, as well as a distribution that reflected racial and ethnic compositions of the counties. Based on these targets, the demographic breakdown of the sample was largely representative.

To help to clarify our sampling strategy, here is how decisions were made about interview locations and potential respondents for interview selection. For example, Brazoria County, with its total city populations of approximately 167,000, represented about 5% of the total number of persons based on the 3.5-million-person FEMA estimate of persons that had been directly or indirectly impacted by the storm. Representing approximately 5% of the total interviews, we estimated 14 interviews would need to be secured from the cities within Brazoria County if we were keeping with our proposed target of 300–350 completed interviews. Alvin, Lake Jackson, and Pearland were specific city targets that we were focusing on in Brazoria County, though interviews came from persons living elsewhere in the county and outside of those city limits. In addition, we added other requirements with regards to which 14 persons could be selected for interviews. First, we had to ensure a reasonable gender distribution (preferably 50:50), as well as a distribution that reflected the racial and ethnic composition of the counties that we were focusing on. To simplify matters, we focused on obtaining White vs. nonWhite interviews, and then once we determined the concentration of Hispanics in each one of the targeted cities, we included that into our final computations of how many nonwhite interviews we would need to target. Again, in the Brazoria County example, where 88% of the county was White, the targets would be 9 white respondents, leaving the remaining 5 interviews to be nonWhite and since 30% of Brazoria County was Hispanic that would mean of the 5 nonwhite target interviews, (2) interviews would need to be Hispanic. We targeted 7 males and 7 females.

Here is how things actually worked when it came to interviewee selection. The data that was collected for Brazoria County included 25 total interviews (our original target was a minimum of 14). The percentage of women was 60% (the original target was 50%). The racial and ethnic targets were pretty precise; 88% of interviews were White which was the current percentage of White residents in Brazoria County. We needed at least a third of nonWhite respondents to be Hispanic and we managed to get 21% of Hispanic interviews. Finally, interviews were divided into groups: Those not having to move from their residence (58%) and the remaining respondents who were displaced (42%), divided across the other displacement options. Keep in mind that these represented targeted estimates, and, in some cases, we were successful in reaching the targets, in other cases, we were not. A similar strategy was used for the collection of the online survey responses. We invoked strict parameters for participation and if persons fit in the pre-determined quotas they were allowed to participate in the survey. Appendix A (Table A1) provides an overview of county demographic estimates and actual completed targeted surveys. While not perfect, the final sample clearly does an adequate job of reflecting the socio-demographic composition of the targeted counties that were most impacted based on our original assumption concerning FEMA targets.

Once the sampling design was completed, several strategies were used to obtain this sample. The first approach was to recruit persons for face-to-face interviews. We did this by contacting local shelters, area hotels/motels receiving vouchers from FEMA, homeless service providers, as well as those that had been interviewed who could provide snowball-like recommendations of friends and family that might meet eligibility requirements for interviewing. This amalgamation of processes led to the completion of nearly one hundred face-to-face interviews. The second approach utilized an online platform (Qualtrics) for digital survey distribution. Qualtrics used our survey that was being used in face-to-face interviewing and built a series of selection protocol questions requiring persons to meet specific criteria prior to their participation. Qualtrics enrolled potential respondents living in targeted zip codes that were part of FEMA’s county estimates receiving the highest levels of damage. Using a set of screening profiles developed to ensure some degree of representativeness, panels of respondents were recruited based on responses to a series of sociodemographic questions (gender, race, Hispanic origin, mover vs. stayer during hurricane). This second strategy netted over 200 completed surveys, yielding a final sample size of 316.

### 3.2. Measurement

#### 3.2.1. Food Insecurity

The dependent variable of interest for the current analysis was *food insecurity*. We measured food insecurity using a two-item screener, coded as 1 = food insecure and 0 = food secure, developed by Hager et al. [55]. These items were selected to capture the key dimensions of food insecurity, while reducing respondent burden on individuals already experiencing an already frustrating and stressful set event. Specifically, individuals were asked “Thinking about your experiences with food, tell us how true the following statements were for you and or your household, (a) I was worried whether my food would run out by the end of the month; (b) the food that I bought just didn’t last, and I didn’t have money to get more.” Responses that affirmed (e.g., somewhat true or very true) an experience of food insecurity were coded 1, while those that indicated food security (e.g., not true at all) were coded as 0. Any respondent who gave an affirmative response to either question was coded as food insecure (1), while those who did not respond affirmatively to either question were coded as food secure (0).

#### 3.2.2. Social Vulnerability

Social vulnerability variables provide some insight regarding how food insecurity vulnerability varies across groups. A number of sociodemographic variables were assessed that have been used in previous research examining the relationship between food insecurity and disaster among adult survivors [15,16,17]. These variables included age, sex, low income, race, and households with children. Age was coded in years. Gender was coded (male/female) with 1 = female. Low income was coded (persons reporting more/less than $20,000 in household earnings) with 1 = < $20,000 annual household income; race was coded as 1 = nonWhite and/or Hispanic to indicate minority race/ethnic status within the US context; and households with children was coded 1 = Yes.

#### 3.2.3. Circumstantial Risk

Circumstantial risk provides an additional assessment of the impact that context plays in shaping food insecurity outcomes. While previous research does not define displacement as a circumstantial control, multiple studies have utilized displacement as a variable of interest in understanding the complexities of post-disaster survival and recovery [35,56,57,58,59,60]. For our purposes, displacement pathways were defined as four outcomes depending on whether survivors: (a) Stayed home; (b) stayed with a friend or relative; (c) stayed in a hotel or motel; and (d) stayed in a shelter and/or were homeless. The largest proportion of our sample stayed home (55%), followed by 20% reporting staying in a shelter or becoming homeless, 15% stayed with a friend or relative, and 5% stayed in a hotel or motel. Because over half the sample reported they stayed home, we constructed a dichotomous displacement variable with persons who left their residence before or during the storm = 1 and those who stayed = 0. In addition we include residence status as a circumstantial risk variable and it is coded dichotomously as rent = 1 and homeowner = 0.

### 3.3. Social and Psychological Resources

#### 3.3.1. Mastery of Fate

Mastery is assessed using a 7-item Likert scale that asks respondents about their ability to control their environment. We used a scale developed by Pearlin and Schooler [43], where higher scores indicate greater mastery and internal locus of control. Scores range from 7–28, with responses ranging from 1 (strongly disagree) to 4 (strongly agree). For the current sample, the scale is modestly reliable with a Cronbach’s alpha = 0.65.

#### 3.3.2. Social Ties

The strength of social ties acts as a resource and potential mitigator of the stress and potential risk caused by living through a disaster. Participants were asked how often they had felt bothered by three problems: (1) having no close companion, (2) not having enough friendships, and (3) not seeing enough people that you feel close to. To measure strength of social ties, we created the following scale based on the item responses: 1 = most or all of the time, 2 = occasionally or a moderate amount of time; 3 =some or a little of the time; 4 = rarely; and 5 = never, with higher scores indicating that respondents had no problems with their social relationships. The variable was reliable with a Cronbach’s alpha = 0.85.

#### 3.3.3. Community Connectedness

We measured connectedness using the Inclusion of Community in Self (ICS) scale, which is a single-item picture measure that consists of six pairs of overlapping circles. This measure, as an extension and variation of the Inclusion of Others in Self Scale (IOS) [61] and a Psychological Sense of Community (PSOC) [49] seems appropriate in the present context for studying disaster survivors and their degree of connectedness to community.

**Figure 1:**
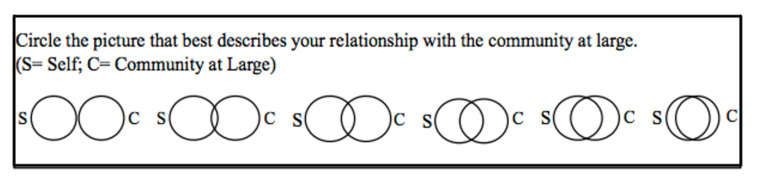
Inclusion of Community in the Self Scale.

As seen in Figure 1, the ICS scale displays two circles of equal size-one circle represents the “self” and the other circle “community.” The first picture in the figure shows two circles that are not touching one another. Subsequent pictures in the figure, moving left to right, display the circles with a varying degree of closeness. The final set of circles was fully integrated with one circle essentially inside that of the other circle. Participants are asked to look at the Venn diagrams and respond with a number associated with a particular circle set that best describes their relationship to the community at large.

With little or no additional explanation provided to interviewees, the majority of respondents appeared to have little difficulty responding to the purposely vague construct of “community at large.” No specific group or subgroup was used as a referent for the face-to-face interviews or online surveys.

### 3.4. Analytical Strategy

We utilize logistic regression analyses to examine relationships between individual variables and food insecurity as well as sets of conceptually related variables (e.g., vulnerabilities, risks, and resources). The decision to use logistic rather than ordinary-least squares regression was made primarily because of the non-linearity of food insecurity and several other variables in the analysis. Finally, in an effort to look more carefully at the circumstantial risk relationships between ownership and displacement, we examined the interaction between these two variables. While typically this interaction term would be included along with the two variables from which it is composed, significant multicollinearity issues arise in such a model. Thus, we present two full models, one with no interaction effects, and a separate one including the interactions with the individual variables (main effects) of residence and displacement removed.

## 4. Results

Characteristics of the sample of Hurricane Harvey survivors are reported in Table 1. Over half the sample (54%) experienced some level of food insecurity. A little more than half (52%) of the respondents that participated in this survey were female. The average age of the sample was approximately 42 years old. Forty-eight percent of the respondents were either Hispanic or non-White, about 21% were reporting have earned less than $20,000 in household income, and 51% reported being in a household with children under the age of 18. Beyond these sociodemographic characteristics, about 42% of the respondents were renting their place at the time of the hurricane. Displacement was reported by approximately 42% of those interviewed, even though nearly three-quarters of the sample reported that they had experienced damage to the structure they were living in that could be characterized from mild to a total loss. The average social ties score was 10.49. The average mastery of fate score was 17.48. The average community connectedness score was 3.1.

The logistic regression models in Table 2, portray a sample of survivors reporting food insecurity in part because of their structural circumstance and background, but also because of specific individual risks and resources. In the first model, with no interaction term included, race/ethnicity, displacement (leaving home), and renting are associated with higher odds of food insecurity. Specifically, racial/ethnic minorities (i.e., Hispanic or non-White respondents) had nearly double the odds of food insecurity (OR = 1.9) compared to those in the dominant racial/ethnic group (i.e., non-Hispanic whites). Those who were displaced, or left their home, also had nearly double the odds of food insecurity (OR =1.9) compared to those who stayed in their home. Renters were more than three times as likely (OR = 3.1) to experience food insecurity than homeowners. Finally, odds of food insecurity were reduced as age increased (OR = 0.96). These results suggest that food insecurity in the post-disaster setting is associated with both social vulnerabilities and circumstantial risks. Strength of social ties, mastery of fate, and community connectedness are associated with lower odds of food insecurity. Specifically, a one-unit increase in the scores for social ties, mastery, and connectedness, were associated with decreased odds of food insecurity by 16%, 20%, and 25%, respectively. These results suggest that psychosocial resources also play an important role in mitigating the odds of food insecurity in a post-disaster setting. Having children in the household and gender did not meet statistical significance standards (two-tailed; *p* < 0.05) in their relationship to food insecurity odds; however, we note the association between sex and food insecurity was very close to this threshold for significance. If we had hypothesized specifically that women would be more food insecure than men and utilized a one-tailed test for significance, the *p*-value would meet this threshold. It is worth noting this since the odds ratio is 1.93, suggesting higher odds of food insecurity experienced by women in this setting compared to men. The main effects model was significant with a *X*^2^ = 113.646 (*p* < 0.001) and a pseudo-R^2^ estimate = 0.327.

In an effort to model the complexity of the relationship between displacement and home ownership, we examine the exact same model, but now with the inclusion of an interaction effects between these two variables. First, it is worth noting that the results for all the other independent variables remain the same regardless of whether the interaction term or their individual variables are included in the model. The more interesting finding, though, is what the interaction term reveals. This term allows us to compare the reference group of those survivors who stayed and owned their home, to those who stayed and rented, those who left and own a home, and those who left and rented their home. We find that the effect of leaving or displacement is moderated by homeownership. Specifically, those who own their home and leave do not have higher odds of food insecurity when compared to other homeowners who stay. Additionally, those persons who rent; however, are worse off regardless of whether they leave or stay in their residence during the storm. Those who rent and stay are over three times (OR = 3.31; *p* < 0.01) as likely to experience food insecurity compared to those who own their home and stay, and renters who leave are nearly six times (OR = 5.96; *p* < 0.001) as likely to be food insecure compared to homeowners who remained home.

## 5. Discussion

The results of this study demonstrate that the odds of experiencing food insecurity in this post-disaster setting were not uniform, but unequal across some social groups, and further shaped by sets of risks and resources rooted in social structure. As Klinenberg [10] argues, disasters are critical tests for governments and societies, and their social protections against the suffering and physical damage that we often assume to be “natural” outcomes associated with such events. Disasters bring with them their own unique challenges, but they also reveal vulnerabilities and protections that existed long before high winds or extreme temperatures take their toll. The Texas Gulf Coast that was devastated by Hurricane Harvey was vulnerable to high food insecurity rates even before the disaster hit, with Harris County having the second highest number of food insecure individuals in the state [12]. Using a “social autopsy” lens provides a glimpse into the ways in which social conditions place some at higher/lower risk of food insecurity in the post-disaster setting and is critical to building the kinds of social protection systems necessary to limit suffering as effectively and justly as possible.

Although some damage following extreme weather events may be natural, the uneven burden and suffering rooted in social structure certainly is not. This study highlights several such inequalities in the burden of this disaster as it relates to food insecurity. In particular, we reveal that age, race, housing, and psychosocial resources all play a role in determining the odds of whether someone will face the added burden of food insecurity amidst a disaster like Hurricane Harvey. These findings are generally consistent with extant research on food insecurity predictors in non-disaster settings [1,7,62,63]. Of specific interest; however, are the ways in which homeownership intersects with the decision to stay or leave ones’ residence, and the impact this has on determining post-disaster food insecurity odds. Our findings highlight that, while leaving may pose its own risks for food insecurity, homeowners are largely protected from that added risk. Furthermore, the decision to stay or leave may be more complicated for renters whose ability to stay or leave may depend more on decisions made by their landlords than their own assessment of what is best for themselves or their family [21,38]. Thus, it is unsurprising that renters who stay and renters who leave both had higher odds of food insecurity than homeowners who stayed. That said, our results also suggest that the highest risk for food insecurity was among renters who were displaced or left their home. There are several possible interpretations of this finding. First, it could be indicative of the challenges that those who leave are faced with when trying to find new, affordable living. Second, it could indicate something about the distribution of rental properties in flood zones compared to owned homes, given that development in Houston has largely ignored the risk of flooding as housing expands in the city [21]. Finally, it could be indicative of the fact that many renters were forced to vacate due to decisions made by their landlords in the aftermath of the disaster, and the general struggles involved in abruptly needing to find new housing regardless of any desire to stay [21,38].

While natural disasters like Hurricane Harvey rightly bring about responses for emergency aid, the social vulnerabilities, risks, and resources we identify in this study highlight the role that long-term social conditions play in shaping the burdens faced by survivors of extreme weather events. Simply put, emergency responses are not sufficient to protect against the uneven and unnecessary suffering that follows such an event. Long-term policy solutions that put in place securities enabling people to endure these events with limited suffering should include those that strengthen the social infrastructure—improving social ties, community connectedness, and housing affordability as well as tenants’ rights. It is not by accident that the vulnerabilities and risks (e.g., renting and exiting) are also conceptually linked to the psychosocial resource variables that can be protective. When people cannot reliably know where they will be in the coming years or even months, building strong social ties, feeling as though your fate is within your own control, and strengthening connections to one’s community become more difficult.

### 5.1. Study Limitations

The current study makes some important contributions to our conceptual and empirical understanding of food insecurity in the disaster setting; however, there are several study limitations that are worth noting. We recognize that a cross-sectional snapshot a few months after a major disaster hits has both positive and negative aspects to the way we report and interpret these findings. Our access was limited, survivors were still in recovery mode, and while the crisis of food insecurity was heightened, it provides only one glimpse into the complicated set of struggles that many face following a disaster. While difficult to execute, long-term longitudinal studies that can assess the struggle to obtain healthy food both prior to and after natural disasters could provide a more controlled view of what food security looks like for survivors and how a whole new group of food insecure individuals emerge during the immediate days and weeks after tragedy strikes.

Additionally, our measures are often limited by the time we had to do the interviews, the access that we had to survivors, and the difficult choices we had to make regarding what measure to use, and how to use it. Ideally, we would have preferred to use multiple measures to assess risks and resources beyond what we developed. While we recognize that more is better, we nevertheless identified and selected key indicators that proved both valid and reliable in the current study. Despite the limits to those indicators, we are able to provide some important insights into the heightened risk and value of social resources during the difficult time immediately following a natural disaster like Hurricane Harvey.

Finally, while our intent was to provide a representative sample of survivors, there were limits to who we could access, how we could access them, and both physical and social constraints that hindered who became part of the final sample. While we made considerable effort to gather both a larger and more representative sample, circumstances prevented carrying out those plans and thus it is important that we exercise some caution with regards to the generalizability of our findings. Nevertheless, our sample represents one of only a few collections of responses from survivors that were close to ground zero in less than a couple of months after Hurricane Harvey made landfall.

### 5.2. Practical Implications

Emergency managers, public health officials, disaster mental health professionals, and volunteer organizations could benefit from the current study’s findings by helping to identify populations that might be susceptible to food insecurity in the post-disaster landscape. Specifically, pre-disaster interventions could identify individuals who are at risk of food insecurity and invite them to participate in community discussions of disaster risk and planning. While communities may identify local factors contributing to food insecurity, our study revealed that, in general, individuals who rented their homes and had less than a high school diploma were more susceptible to disaster food insecurity when compared to their counterparts. Taken together, these individual risk and social vulnerability factors represent a portion of disaster-affected populations known as “vulnerability bearers” [64,65], and they should be included in broader discussions of community needs. While the inclusion of ‘vulnerability bearers’ in community discussions of disaster risk and planning raises awareness of their lived experiences so as to shape local policy and strategy, such intervention also serves to facilitate community connectedness, which our study revealed to be a significant resource negatively impacting food insecurity. In particular, residents who may feel disconnected from their communities may also lack linking social capital, which “connects regular citizens with those in power” [54]. By inviting residents who possess the risk and social vulnerability factors of disaster food insecurity to community meetings about disaster planning, this intervention creates a space for groups traditionally left out of policymaking circles to be heard, to challenge pre-existing beliefs about vulnerability bearers as it relates to food security and disasters [66], and to discuss how disasters have the potential to disrupt the food distribution resources they may normally rely upon (e.g., food banks, family). The incorporation of vulnerability bearers into such discussions may also build trust with the Hispanic community, which has been found to have lower expectations that government and disaster relief services will provide post-disaster support [67]. Furthermore, efforts should be made to implement interventions that address risks and bolster social resources particularly for those at-risk subpopulations.

Residents who are food insecure in urban communities are more likely than their rural counterparts to access resources from food distributors like food banks, food assistance programs, and community meal sites [68] that may be overwhelmed or disrupted immediately following disasters. In urban settings, interventions may focus on establishing networks of reciprocity through community currency or time bank programs where individuals offer goods, services, or labor, earn credits, and then exchange those credits for other goods, services, or labor. One of the most frequently used services in time banking programs is food preparation and delivery [69], and participation in time banks has been found to build a sense of community to the program particularly among individuals who are older, less educated, and have a lower SES [70].

While we provide some important early observations regarding Hurricane Harvey survivors’ unequal exposure to disaster and its impact on food insecurity, additional health complications muddy the recovery picture for the vulnerability bearers. These vulnerability bearers are found to have significant physical and mental health complications as a direct result of unequal exposure to trauma that is partly related to who they are as well as where they live [71]. These health complications create a complex intersection of suffering that can further impact levels of food security. Lessons learned are noteworthy, but if we do not heed the warnings and change our pre-disaster preparation, Katrina, Harvey, Maria and the others making up this long list of recent disasters serve not as reminders of what needs to be done but rather reminders of what we still cannot seem to figure out when it comes to being ready and serving the disenfranchised and disadvantaged living in these high-risk disaster zones.

## Figures and Tables

**Table 1:** Descriptive statistics for model variables.

	%	Mean	S.D.
Dependent Variable			
Food Insecurity (1 = Insecure)	54.5	--	0.49
Social Vulnerabilities			
Age (18–80)	--	41.9	14.9
Gender (1 = Female)	52.8	--	0.49
Minority (1 = Hispanic and/or non-White)	48.3	--	0.50
Low Income (1 = Less than $20K)	21.2	--	0.41
Households w/Children (1 = Yes)	51.9	--	0.50
Circumstantial Risks			
Pathway (1 = Left)	42.1	--	0.49
Residence (1 = Renter)	42.5	--	0.43
Social and Psychological Resources			
Strength of Social Ties Scale (3–15)	--	10.5	3.8
Mastery of Fate (7–27)	--	17.5	3.4
Community Connectedness (1–6)	--	3.1	1.6

**Table 2:** Logistic regressions for food insecurity, with and without renter X displacement interactions.

	No Interaction				Interaction		
	OR	*p*	C.I.		OR	*p*	C.I.
Social Vulnerabilities							
Age	0.964	0.004 **	0.940–0.988		0.964	0.004 **	0.940-0.988
Sex (1 = Female)	1.928	0.055	0.986–3.77	Sex	1.915	0.059	0.977–3.75
Race (1 = Non-White or Hispanic)	1.975	0.048 *	1.01–3.88	Race	1.997	0.047 *	1.01–3.95
Low income (1 = Less than $20K)	1.992	0.160	0.761–5.21	Low Income	1.991	0.161	0.760–5.21
Household w/ children (1 = 1 or more)	0.887	0.738	0.441–1.79	HH w/children	0.881	0.724	0.436–1.78
Circumstantial Risks							
Pathway (1 = Left home)	1.959	0.050 *	1.00–3.84	Stayed/Owner	1		
Residence (1 = Renter)	3.119	0.001 ***	1.59–6.11	Stayed/Renter	3.316	0.007 **	1.39–7.87
				Left/Owner	2.091	0.104	0.86–5.08
				Left/Renter	5.96	0.001 ***	2.11–16.82
Social and Psychological Resources							
Strength of Social Ties	0.848	0.000 ***	0.774–0.929	Strength of Social Ties	0.847	0.000 ***	0.773–0.929
Mastery of Fate	0.802	0.000 ***	0.717–0.897	Mastery of fate	0.803	0.000 ***	0.717–0.898
Community Connectedness	0.751	0.011 *	0.602–0.937	Comm. Connectedness	0.754	0.013 *	0.603–0.942
Constant	699.96	0.000		Constant	660.83	0.000 ***	
Pseudo r-squared	0.327			Pseudo r-squared	0.327		
*n*	251.00			*n*	251.00		
Prob > chi2	0.000			Prob > chi2	0.000		

*** *p* < 0.001, ** *p* < 0.01, * *p* < 0.05; OR = Odd Ratio.

## Appendix A

**Table A1:** Demographic breakdown for sampled counties ^1^.

	Harris	Jefferson	Aransas	Galveston	Nueces	Brazoria	County Average
Total Population	4,589,928	254,679	25,721	329,431	361,350	354,195	---
%Male	49.6%	51.1%	49.5%	49.5%	49.4%	50.5%	49.9%
%White	69.6%	59.1%	92.9%	80.3%	90.9%	74.5%	77.9%
%Hispanic	43.7%	22.1%	27.9%	25.4%	64.5%	31.6%	35.9%
%African American	20.0%	34.1%	1.7%	13.2%	4.3%	15.1%	14.7%

^1^ Sociodemographic composition of sampled counties on Texas Gulf Coast 2017 used to develop sampling targets. Available online: https://census.gov (accessed online 2 November 2020).

## References

[1] Coleman-Jensen A., Rabbitt M.P., Gregory C.A., Singh A. (2019). Household Food Security in the United States in 2018.

[2] Pollard C.M., Booth S. (2019). Food Insecurity and Hunger in Rich Countries—It Is Time for Action against Inequality. Int. J. Environ. Res. Public Health.

[3] Scanlan S.J. (2009). New Direction and Discovery on the Hunger Front: Toward a Sociology of Food Security/Insecurity. Humanit. Soc..

[4] Sen A. (1981). Poverty and Famines: An Essay on Entitlement and Deprivation.

[5] Carolan M.S. (2014). Reclaiming Food Security. J. Int. Aff..

[6] Arteaga I., Potochnick S., Parsons S. (2017). Decomposing the Household Food Insecurity Gap for Children of U.S.-Born and Foreign-Born Hispanics: Evidence from 1998 to 2011. J. Immigr. Minority Health.

[7] Lee B.A., Greif M.J. (2008). Homelessness and hunger. J. Health Soc. Behav..

[8] Holland A.C., Kennedy M.C., Hwang S.W. (2011). The assessment of food security in homeless individuals: A comparison of the Food Security Survey Module and the Household Food Insecurity Access Scale. Public Health Nutr..

[9] Martin M.A., Lippert A.M. (2012). Feeding her children, but risking her health: The intersection of gender, household food insecurity and obesity. Soc. Sci. Med..

[10] Klinenberg E. (2002). Heat Wave: A Social Autopsy of Disaster in Chicago.

[11] Tierney K. (2014). The Social Roots of Risk: Producing Disasters, Promoting Resilience.

[12] Feeding America (2017). 7 Million Hurricane-Impacted People Were Food Insecure.

[13] World Central Kitchen. https://wck.org.

[14] Tierney K.J., Lindell M.K., Perry R.W. (2001). Facing the Unexpected: Disaster Preparedness and Response in the United States.

[15] Clay L.A., Ross A.D. (2020). Factors Associated with Food Insecurity Following Hurricane Harvey in Texas. Int. J. Environ. Res. Public Health.

[16] Clay L.A., Papas M.A., Gill K.B., Abramson D.M. (2018). Application of a Theoretical Model Toward Understanding Continued Food Insecurity Post Hurricane Katrina. Disaster Med. Public Health Prep..

[17] Clay L.A., Papas M.A., Gill K.B., Abramson D.M. (2018). Factors Associated with Continued Food Insecurity among Households Recovering from Hurricane Katrina. Int. J. Environ. Res. Public Health.

[18] MacNabb E., Fletcher B.J., Rivera F. (2019). Hurricanes, Disasters, and Food Insecurity. Emerging Voices in Natural Hazards Research.

[19] Blake E.S., Zelinsky D.A. (2018). National Hurricane Center Tropical Cyclone Report: Hurricane Harvey.

[20] Fritz A., Samenow J. (2017). Harvey unloaded 33 trillion gallons of water in the U.S. The Washington Post.

[21] Dickerson M. (2018). Hurricane Harvey and the Houston Housing Market. Tex. Law Rev..

[22] Fabiano J. Timeline Recounts the Devastating 2017 Atlantic Hurricane Season and Storms that Made it Memorable.

[23] Balbus J.M., Malina C. (2009). Identifying vulnerable subpopulations for climate change health effects in the United States. J. Occup. Environ. Med..

[24] Pyles L., Kulkarni S., Lein L. (2008). Economic Survival Strategies and Food Insecurity. J. Soc. Serv. Res..

[25] Fitzpatrick K., LaGory M. (2013). Unhealthy Cities: Poverty, Race, and Place in America.

[26] Hoffman J.S., Shandas V., Pendleton N. (2020). The Effects of Historical Housing Policies on Resident Exposure to Intra-Urban Heat: A Study of 108 US Urban Areas. Climate.

[27] Klinenberg E. (2001). Dying alone: The social production of urban isolation. Ethnography.

[28] Klinenberg E. (2016). Social Isolation, Loneliness, and Living Alone: Identifying the Risks for Public Health. Am. J. Public Health.

[29] Ueland J., Warf B. (2006). Racialized Topographies: Altitude and Race in Southern Cities. Geogr. Rev..

[30] Chakraborty J., Collins T.W., Grineski S.E. (2018). Exploring the Environmental Justice Implications of Hurricane Harvey Flooding in Greater Houston, Texas. Am. J. Public Health.

[31] Hamel L., Wu B., Brodie M., Sim S.-C., Marks E. (2017). An Early Assessment of Hurricane Harvey’s Impact on Vulnerable Texans in the Gulf Coast Region: Their Voices and Priorities to Inform Rebuilding Efforts.

[32] Heimlich R. (2012). Houston Tops the List of Major Metro Areas in Economic Segregation by Income.

[33] Masozera M., Bailey M., Kerchner C. (2007). Distribution of impacts of natural disasters across income groups: A case study of New Orleans. Ecol. Econ..

[34] Neumayer E., Plümper T. (2007). The Gendered Nature of Natural Disasters: The Impact of Catastrophic Events on the Gender Gap in Life Expectancy, 1981–2002. Ann. Assoc. Am. Geogr..

[35] Lowe S.R., Tracy M., Cerdá M., Norris F.H., Galea S. (2013). Immediate and longer-term stressors and the mental health of Hurricane Ike survivors. J. Trauma. Stress.

[36] Uscher-Pines L. (2009). Health effects of relocation following disaster: A systematic review of the literature. Disasters.

[37] Desmond M. (2016). Evicted: Poverty and Profit in the American City.

[38] Smith M. (2017). For Renters, Harvey Was the First Blow, Followed by Orders to Move (Published 2017). The New York Times.

[39] Lin N., Dumin M. (1986). Access to occupations through social ties. Soc. Netw..

[40] Lin N., Ensel W.M. (1989). Life stress and health: Stressors and resources. Am. Sociol. Rev..

[41] Pearlin L.I. (1989). The sociological study of stress. J. Health Soc. Behav..

[42] Pearlin L.I., Menaghan E.G., Lieberman M.A., Mullan J.T. (1981). The stress process. J. Health Soc. Behav..

[43] Pearlin L.I., Schooler C. (1978). The structure of coping. J. Health Soc. Behav..

[44] Thoits P.A. (1995). Stress, coping, and social support processes: Where are we? What next?. J. Health Soc. Behav..

[45] Thoits P.A. (2010). Stress and health: Major findings and policy implications. J. Health Soc. Behav..

[46] Thoits P.A. (2006). Personal agency in the stress process. J. Health Soc. Behav..

[47] Wheaton B. (1983). Stress, Personal Coping Resources, and Psychiatric Symptoms: An Investigation of Interactive Models. J. Health Soc. Behav..

[48] Wheaton B. (1985). Models for the stress-buffering functions of coping resources. J. Health Soc. Behav..

[49] McMillan D.W., Chavis D.M. (1986). Sense of community: A definition and theory. J. Community Psychol..

[50] Bourdieu P. (1986). The forms of capital. Handbook of Theory and Research for the Sociology of Education.

[51] Dean W.R., Sharkey J.R. (2011). Food insecurity, social capital and perceived personal disparity in a predominantly rural region of Texas: An individual-level analysis. Soc. Sci. Med..

[52] Martin K.S., Rogers B.L., Cook J.T., Joseph H.M. (2004). Social capital is associated with decreased risk of hunger. Soc. Sci. Med..

[53] Willis D.E., Fitzpatrick K.M. (2017). Food insecurity and social capital among middle school students. Youth Soc..

[54] Aldrich D.P., Meyer M.A. (2015). Social Capital and Community Resilience. Am. Behav. Sci..

[55] Hager E.R., Quigg A.M., Black M.M., Coleman S.M., Heeren T., Rose-Jacobs R., Cook J.T., de Cuba S.A.E., Casey P.H., Chilton M. (2010). Development and Validity of a 2-Item Screen to Identify Families at Risk for Food Insecurity. Pediatrics.

[56] Acierno R., Ruggiero K.J., Kilpatrick D.G., Resnick H.S., Galea S. (2006). Risk and protective factors for psychopathology among older versus younger adults after the 2004 Florida hurricanes. Am. J. Geriatr. Psychiatry.

[57] DeSalvo K.B., Hyre A.D., Ompad D.C., Menke A., Tynes L.L., Muntner P. (2007). Symptoms of Posttraumatic Stress Disorder in a New Orleans Workforce Following Hurricane Katrina. J. Urban Health.

[58] Lowe S.R., Sampson L., Gruebner O., Galea S. (2015). Psychological resilience after Hurricane Sandy: The influence of individual- and community-level factors on mental health after a large-scale natural disaster. PLoS ONE.

[59] Lowe S.R., Sampson L., Gruebner O., Galea S. (2016). Community Unemployment and Disaster-Related Stressors Shape Risk for Posttraumatic Stress in the Longer-Term Aftermath of Hurricane Sandy. J. Trauma Stress.

[60] Tracy M., Norris F.H., Galea S. (2011). Differences in the determinants of posttraumatic stress disorder and depression after a mass traumatic event. Depress. Anxiety.

[61] Aron A., Aron E.N., Smollan D. (1992). Inclusion of other in the self scale and the structure of interpersonal closeness. J. Personal. Soc. Psychol..

[62] Willis D.E. (2020). Feeding inequality: Food insecurity, social status and college student health. Sociol. Health Illness.

[63] Willis D.E., Fitzpatrick K.M. (2016). Psychosocial factors as mediators of food insecurity and weight status among middle school students. Appetite.

[64] Krajeski R. (2018). Framing Research: Concepts and Jargon in Disaster Research.

[65] Peek L. (2019). The Vulnerability Bearers.

[66] Dutta M.I., Anaele A., Jones C. (2013). Voices of hunger: Addressing health disparities through the culture-centered approach. J. Commun..

[67] Messias D.K., Hilfinger C.B., Lacy E. (2013). Latino social network dynamics and the hurricane Katrina disaster. Disasters.

[68] Morton L.W., Bitto E.A., Oakland M.J., Sand M. (2008). Accessing food resources: Rural and urban patterns of giving and getting food. Agric. Hum. Values.

[69] Collom E., Lasker J.N. (2016). Equal Time, Equal Value: Community Currencies and Time Banking in the US.

[70] Lasker J., Collom E., Bealer T., Niclaus E., Young Keefe J., Kratzer Z., Baldasari L., Kramer E., Mandeville R., Schulman J. (2011). Time banking and health: The role of a community currency organization in enhancing well-being. Health Promot. Pract..

[71] Fitzpatrick K.M., Spialek M.L. (2020). Hurricane Harvey’s Aftermath: Place, Race, and Inequality in Disaster Recovery.

